# The altered expression of autophagy-related genes participates in heart failure: *NRBP2* and *CALCOCO2* are associated with left ventricular dysfunction parameters in human dilated cardiomyopathy

**DOI:** 10.1371/journal.pone.0215818

**Published:** 2019-04-22

**Authors:** Carolina Gil-Cayuela, Alejandro López, Luis Martínez-Dolz, José Ramón González-Juanatey, Francisca Lago, Esther Roselló-Lletí, Miguel Rivera, Manuel Portolés

**Affiliations:** 1 Cardiocirculatory Unit, Health Research Institute of La Fe University Hospital (IIS La Fe), Valencia, Spain; 2 Heart Failure and Transplantation Unit, Cardiology Department, La Fe University Hospital, Valencia, Spain; 3 Cellular and Molecular Cardiology Research Unit, Department of Cardiology and Institute of Biomedical Research, University Clinical Hospital, Santiago de Compostela, Spain; Cincinnati Children's Hospital Medical Center, UNITED STATES

## Abstract

This study aimed to analyze changes in the expression of autophagy- and phagocytosis-related genes in patients with dilated cardiomyopathy (DCM), especially in relation to left ventricular (LV) dysfunction. Furthermore, transmission electron microscopy of the diseased tissue was carried out to investigate if the gene expression changes are translated into ultrastructural alterations. LV tissue samples from patients with DCM (n = 13) and from controls (CNT; n = 10) were analyzed by RNA-sequencing, whereupon the altered expression (*P* < 0.05) of 13 autophagy- and 3 phagocytosis-related genes was observed. The expression changes of the autophagy-related genes *NRBP2* and *CALCOCO2* were associated with cardiac dysfunction and remodeling (*P* < 0.05). The affected patients had a higher activity of these degradation processes, as evidenced by the greater number of autophagic structures in the DCM tissue (*P* < 0.001). Differences in the ultrastructural distribution were also found between the DCM and CNT tissues. These results show that in patients with DCM, the altered expression of *NRBP2* and *CALCOCO2* is related to LV dysfunction and remodeling. Clarification of the molecular mechanisms of cardiac autophagy would help in the future development of therapies to improve LV performance.

## Introduction

Dilated cardiomyopathy (DCM) is characterized by the dilation of the cardiac chambers with increases in the ventricular mass and wall thickness, impaired myocardial contractility, and ventricular dysfunction that frequently results in heart failure. It is a condition that is increasing in prevalence and carries a high mortality rate, with no curative treatment available [1,2]. The development of heart failure is known to be associated with the altered expression of genes involved in the production of various subcellular organelles and structures (e.g., the extracellular matrix [3,4], endoplasmic and sarcoplasmic reticulums [5], and Golgi apparatus [6]), which results in alterations to the cellular processes in which these structures participate. However, there are no published transcriptomic studies focused on the expression levels of genes involved in the processes of autophagy and phagocytosis. These processes play an indispensable role in the degradation of cellular structures. During phagocytosis, cells internalize and enclose surrounding microorganisms or unusable or harmful cell debris through generation of the phagosome, a single-membrane structure that merges with a lysosome to produce a phagolysosome wherein degradation takes place [7]. In autophagy, cytoplasmic components are engulfed by a membrane structure known as the phagophore. This membranous structure enlarges either by the direct inflow of the endoplasmic reticulum or by the addition of vesicles to eventually generate a double-membranous autophagosome that closes off, thereby sequestering the cytosolic material within it [8]. The autophagosome then merges with a lysosome, forming the autophagolysosome that executes the degradation of the autophagosome’s internal contents as well as its inner membrane [7].

In this context, the Golgi apparatus has been described as a potential membrane source for autophagosome development [9], which is an essential stage of the autophagy mechanism. In a previous study on DCM, we reported structural alterations in the Golgi apparatus of patients with this cardiomyopathy as well as changes in the expression of genes related to Golgi function [6]. Given the relevance of these degradation processes in intracellular self-renewal, energy replenishment, and substrate recycling [10], the relationship between the Golgi apparatus and autophagosome development, and the previous results found, we postulated that changes in the expression of autophagy- and phagocytosis-related genes are associated with cardiac dysfunction and remodeling in patients with DCM. To verify this, in the current study, we examined the changes in expression of autophagy- and phagocytosis-related genes in cardiac tissue from patients with DCM compared with those from a control (CNT) group, as well as the impact of such altered gene expression on left ventricular (LV) dysfunction and remodeling. In addition, using transmission electron microscopy (TEM), we searched for potential changes in autophagic structures in the diseased tissue.

## Materials and methods

### Collection of cardiac tissue samples

A total of 23 LV tissue samples were collected from heart explants from patients with DCM (n = 13) undergoing cardiac transplantation, or from deceased donors as controls (CNT, n = 10), for use in the RNA-sequencing (RNA-Seq) analysis. To improve the numerical base with a higher number of patients, we increased the DCM samples to 20 for the protein analysis. As inclusion criteria we only considered non-ischemic idiopathic DCM samples. Non-ischemic DCM was diagnosed when patients showed intact coronary arteries by coronary angiography, and LV systolic dysfunction [ejection fraction (EF) < 40%] with a dilated left ventricle [LV end-diastolic diameter (LVEDD) > 55 mm]. As exclusion criteria, patients with primary valvular disease or familial DCM were not considered for the study. All patients were functionally classified according to the New York Heart Association (NYHA) criteria, and were receiving medical treatment (Table 1) according to the guidelines of the European Society of Cardiology [11].

**Table 1:** Medical treatment of the patients with dilated cardiomyopathy.

Treatment (%)	Patients (n = 20)
Angiotensin-converting enzyme inhibitors	78
Angiotensin II receptor antagonists	6
Beta-blockers	67
Aldosterone antagonists	94
Digoxin	78
Antidiabetics	6
Diuretics	100
Statins	22
Antithrombotics	72
Antiarrhythmics	22

The CNT samples were obtained from non-diseased hearts that could not be transplanted owing to surgical reasons or blood type incompatibility. The cause of death of these donors was a cerebrovascular event or a motor vehicle accident. All CNT hearts showed normal LV function (EF > 50%) as determined by Doppler echocardiography, and had no history of myocardial disease or active infection at the time of transplantation. Fresh transmural samples were obtained from near the apex of the LV at the time of transplantation and preserved in 0.9% NaCl at 4°C for a maximum of 6 h from the time of removal from coronary circulation. The tissue samples were stored at –80°C until use. A reduced time between sample receipt and storage yielded higher-quality samples, as evidenced by the RNA integrity numbers of ≥ 9.

This study was approved by the Ethics Committee (Biomedical Investigation Ethics Committee of La Fe University Hospital, Spain) in accordance with the guidelines of the Declaration of Helsinki [12]. All tissue samples were obtained with the written informed consent of the patients or their close relatives.

### RNA extraction

The cardiac tissue samples were homogenized in TRIzol reagent using the TissueLyser LT system (Qiagen, Manchester, UK). RNA was extracted using the PureLink Kit (Ambion Life Technologies, Carlsbad, CA, USA), following the manufacturer’s instructions. The RNA concentration was measured on the NanoDrop 1000 spectrophotometer (Thermo Fisher Scientific, Leicestershire, UK) and the purity and integrity of the RNA samples were measured using the microfluidics-based 2100 Bioanalyzer platform with the RNA 6000 Nano LabChip Kit (Agilent Technologies, Santa Clara, CA, USA). All RNA samples had a 260/280 absorbance ratio of ≥2.0 and reached a minimal RNA integrity number of ≥9.

### RNA sequencing analysis

Poly(A)-RNA samples were isolated using the MicroPoly(A) Purist Kit (Ambion, Life Technologies, Carlsbad, CA, USA). Total Poly(A)-RNA was used to generate whole-transcriptome libraries for sequencing on the SOLiD 5500XL platform, following the manufacturer’s recommendations (Life Technologies, Carlsbad, CA, USA). The quality of the amplified cDNA was analyzed using the Bioanalyzer 2100 DNA 1000 Kit (Agilent Technologies, Santa Clara, CA, USA) and quantified using the Qubit 2.0 Fluorometer (Invitrogen, Paisley, UK). The whole-transcriptome libraries were used for making SOLiD-templated beads, following the SOLiD templated bead preparation guidelines. The bead quality was assessed on the basis of the workflow analysis parameters. The samples were sequenced using a 50625 paired-end protocol, generating 75 nt + 35 nt (paired-end) + 5 nt (barcode) sequences. The quality of the data was determined using SOLiD experimental tracking software.

### Computational analysis of the RNA sequencing data

The initial whole-transcriptome paired-end reads obtained from sequencing were mapped against the latest version of the human genome (version GRchr37/hg19), using the Life Technologies mapping algorithm (http://www.lifetechnologies.com/, version 1.3). The RNA-Seq computational analysis methods have been extensively described by Gil-Cayuela et al. [13]. The data presented in this manuscript have been deposited in the NCBI’s Gene Expression Omnibus (GEO) database and are accessible through the GEO series accession number GSE55296 (http://www.ncbi.nlm.nih.gov/geo/query/acc.cgi?acc=GSE55296).

### Homogenization of samples, polyacrylamide gel electrophoresis, and western blot analysis

Aliquots (30 mg) of the frozen LV samples were transferred into Lysing Matrix D tubes designed for use with the FastPrep-24 homogenizer (MP Biomedicals, Solon, OH, USA), in total protein extraction buffer (2% SDS, 10 mM EDTA, 6 mM Tris-HCl, pH 7.4) with protease inhibitors (25 μg/mL aprotinin and 10 μg/mL leupeptin). The homogenates were centrifuged and the supernatants were aliquoted. The protein contents of the aliquots were determined according to Peterson’s modification of the micro Lowry method, using bovine serum albumin (BSA) as a standard [14].

For the detection of nuclear receptor-binding protein 2 (NRBP2) and calcium-binding and coiled-coil domain-containing 2 (CALCOCO2), protein samples were first separated by Bis-Tris electrophoresis on a 4–12% polyacrylamide gels under non-reducing conditions and reducing conditions respectively. The bands on the gels were transferred to a polyvinylidene difluoride membrane, using the iBlot Gel Transfer Device (Invitrogen, UK), for western blot analysis. The membranes were first blocked overnight at 4°C with 1% BSA in Tris-buffer solution containing 0.05% Tween-20 and then incubated for 2 h with the primary antibody in the same buffer. The primary antibodies used were anti-NRBP2 rabbit polyclonal antibody (1:2000; ab227480) and anti-CALCOCO2 rabbit polyclonal antibody (1:800; ab68588), both obtained from Abcam (Cambridge, UK); the mouse monoclonal anti-GAPDH antibody (1:800; ab-9484) from Abcam (Cambridge, UK) was used as a loading control. The bands were visualized using an acid phosphatase-conjugated secondary antibody and the nitro-blue tetrazolium/5-bromo-4-chloro-3-indolyl phosphate (Sigma, St. Louis, MO, USA) substrate system. Finally, the bands were digitalized using an image analyzer (DNR Bio-Imagining Systems, Jerusalem, Israel) and quantified by the GelQuant Pro (version 12.2) program.

### Transmission electron microscopy

Pre-fixed tissue samples were washed with 0.1 M cacodylate buffer and incubated in a 2% osmium tetroxide solution for 3 h at 4°C in the dark. The fragments were then incubated in a 2% solution of uranyl acetate for 2 h at room temperature in the dark. This was followed by their dehydration in ketones of increasing concentration (25%, 50%, 75%, 90%, 95%, and 100%) at 4°C. Embedding was carried out by soaking the fragments in successive Epon 812-acetone mixtures (ratios 1:3, 1:1, and 3:1) at 4°C for 1 h in each mixture. Then, three embedments in pure Epon 812 resin were performed at room temperature; the first for 1 h and the other two for 2 h. Finally, one last embedment in Epon 812 was performed at 60°C for at least 48 h.

Ultra-thin sections of approximately 80 nm thickness were obtained by ultramicrotomy (Leica EM UC6) and mounted on copper grids precoated with Formvar film. After 24 h, the sections were stained with 1% uranyl acetate (2% uranyl in absolute ethanol) for 1 min in the dark and then washed with double-distilled water containing 2.7% lead citrate (0.2 M sodium citrate dihydrate, 0.08 M lead nitrate, and 1 M NaOH) for 1 min. Once washed and dried, the gratings were preserved until their analysis by TEM.

A JEOL TEM system (model JEM-1010; at the microscopy service of the Polytechnic University of Valencia) was used with magnifications in the range of 4500×–25000×. Micrographs of the cardiac tissue samples were obtained from systematic uniform random samples of successive sections [15].

### Statistical analysis

Data are expressed as the mean ± standard deviation (SD) for continuous variables and as percentage values for discrete variables. The Kolmogorov–Smirnov test was applied to evaluate the data distribution. All data were normally distributed. Comparisons of clinical characteristics were performed using Student’s *t*-test for continuous variables and Fisher’s exact test for discrete variables. Student’s *t*-test was used to determine significant differences in the mRNA and protein levels between groups. Bivariate correlation analysis was used to study the relationship between the mRNA and protein levels, and the clinical parameters. Pearson’s correlation coefficient was calculated to analyze the association between normal variables. Significance was defined for *P*-values < 0.05. Genes with a fold change of ≥1.3 were considered to be differentially expressed. Statistical analysis was performed using the Statistical Package for Social Sciences, version 20.0 (IBM SPSS, Chicago, IL, USA).

## Results

### Clinical characteristics of the patients

We analyzed a total of 30 LV tissue samples from 20 patients diagnosed with non-ischemic DCM and 10 non-diseased CNT samples. The DCM tissue samples used in the RNA-Seq and protein analyses were mainly from men (92% and 74%, respectively), with a mean age of 51 ± 11 and 47 ± 14 years, respectively. These patients had an NYHA functional classification of III–IV and were previously diagnosed with significant comorbidities (Table 2).

**Table 2:** Clinical characteristics of the patients with dilated cardiomyopathy whose tissues were examined by RNA-Seq and western blot analyses.

	RNA-Seq	Western blotting
	Patients (n = 13)	Patients (n = 20)
Age (years)	51 ± 11	47 ± 14
Gender male (%)	92	74
NYHA class	3.4 ± 0.4	3.3 ± 0.4
BMI (kg/m^2^)	27 ± 5	27 ± 7
Total cholesterol (mg/dL)	147 ± 37	139 ± 49
Prior hypertension (%)	17	19
Prior diabetes mellitus (%)	17	12
Hemoglobin (mg/mL)	13 ± 3	13 ± 3
Hematocrit (%)	39 ± 8	39 ± 6
Smokers (%)	50	53
EF (%)	20 ± 7	22 ± 9
LVESD (mm)	71 ± 12	66 ±12
LVEDD (mm)	80 ± 11	75 ± 11
LVMI (g/m^2^)	241 ± 77	200 ± 46

Data are expressed as the mean value ± standard deviation. RNA-Seq, RNA sequencing; NYHA, New York Heart Association; BMI, body mass index; EF, ejection fraction; LVESD, left ventricular end-systolic diameter; LVEDD, left ventricular end-diastolic diameter; LVMI, left ventricular mass index.

The CNT group also consisted mainly of men (80%), with a mean age of 47 ± 16 years. We did not find significant differences regarding age or gender between both DCM and CNT groups. All CNT hearts showed normal LV function (EF > 50%) as determined by Doppler echocardiography, and had no history of myocardial disease or active infection at the time of transplantation. No other data were available for the CNT group, in accordance with the Spanish Organic Law on Data Protection 15/1999.

### Differentially expressed autophagy- and phagocytosis-related genes

**Fig 1:**
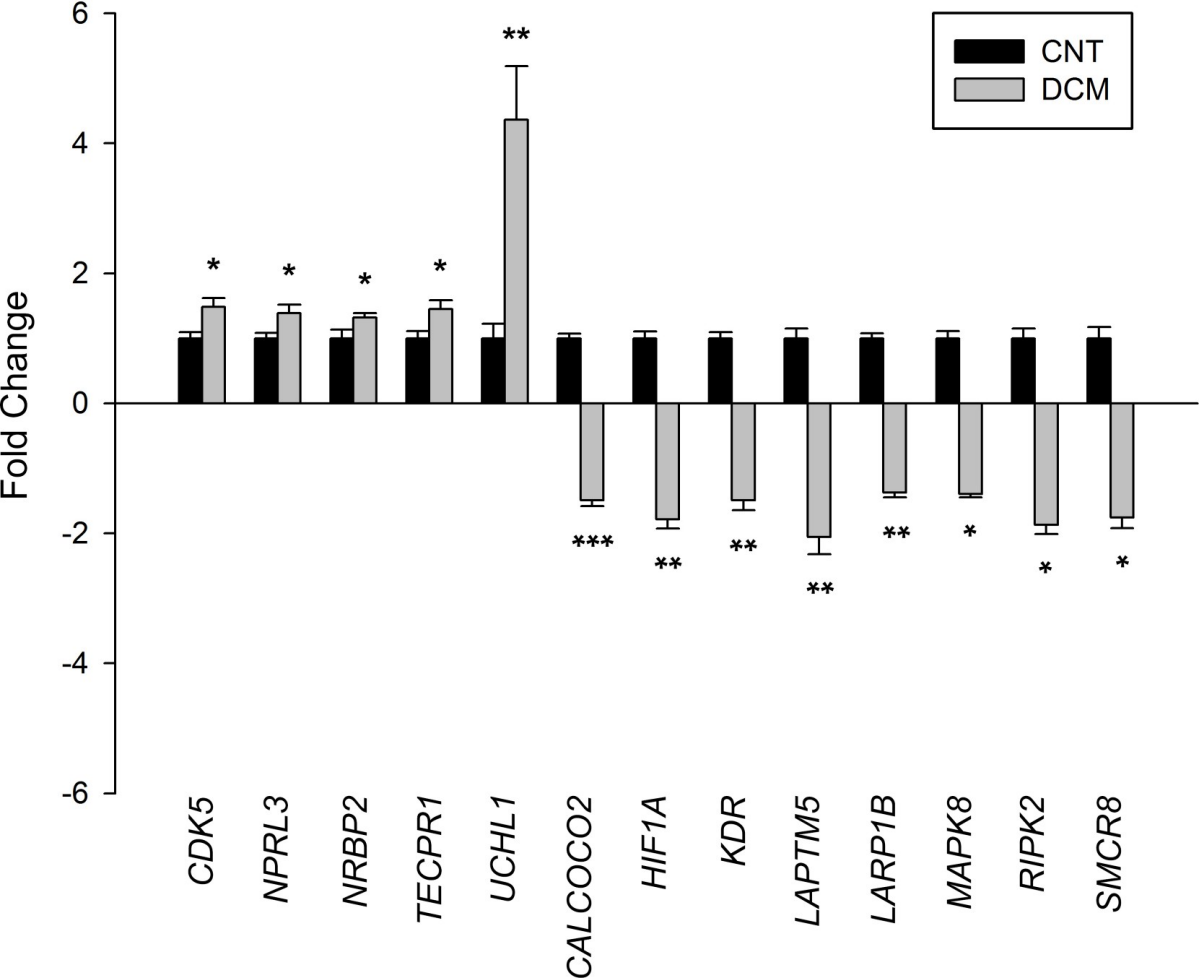
Altered expression of the autophagy machinery in dilated cardiomyopathy. Changes in the mRNA expression of autophagy-related genes in dilated hearts (gray bars) *vs*. control (CNT) hearts (black bars). The values from the CNTs were set to 1. Data are presented as the fold change ± standard error of the mean. *P < 0.05, **P < 0.01, ***P < 0.001 *vs*. CNTs.

To investigate the changes in gene expression between the patients with DCM and the CNT individuals, we performed a large-scale gene expression screening using RNA-Seq technology. We used the Gene Ontology Consortium database to classify the genes involved in the main degradation processes, identifying expression 175 genes involved in the autophagy process (S1 Table), of which 13 genes were differentially expressed (≥1.3-fold, *P* < 0.05) between the DCM and CNT groups. Representative fold-change values of the expression levels of the altered autophagy-related genes are shown in Fig 1. Of these differentially expressed genes, 62% had decreased mRNA expression whereas 38% were upregulated relative to the CNT levels (Fig 1).

**Fig 2:**
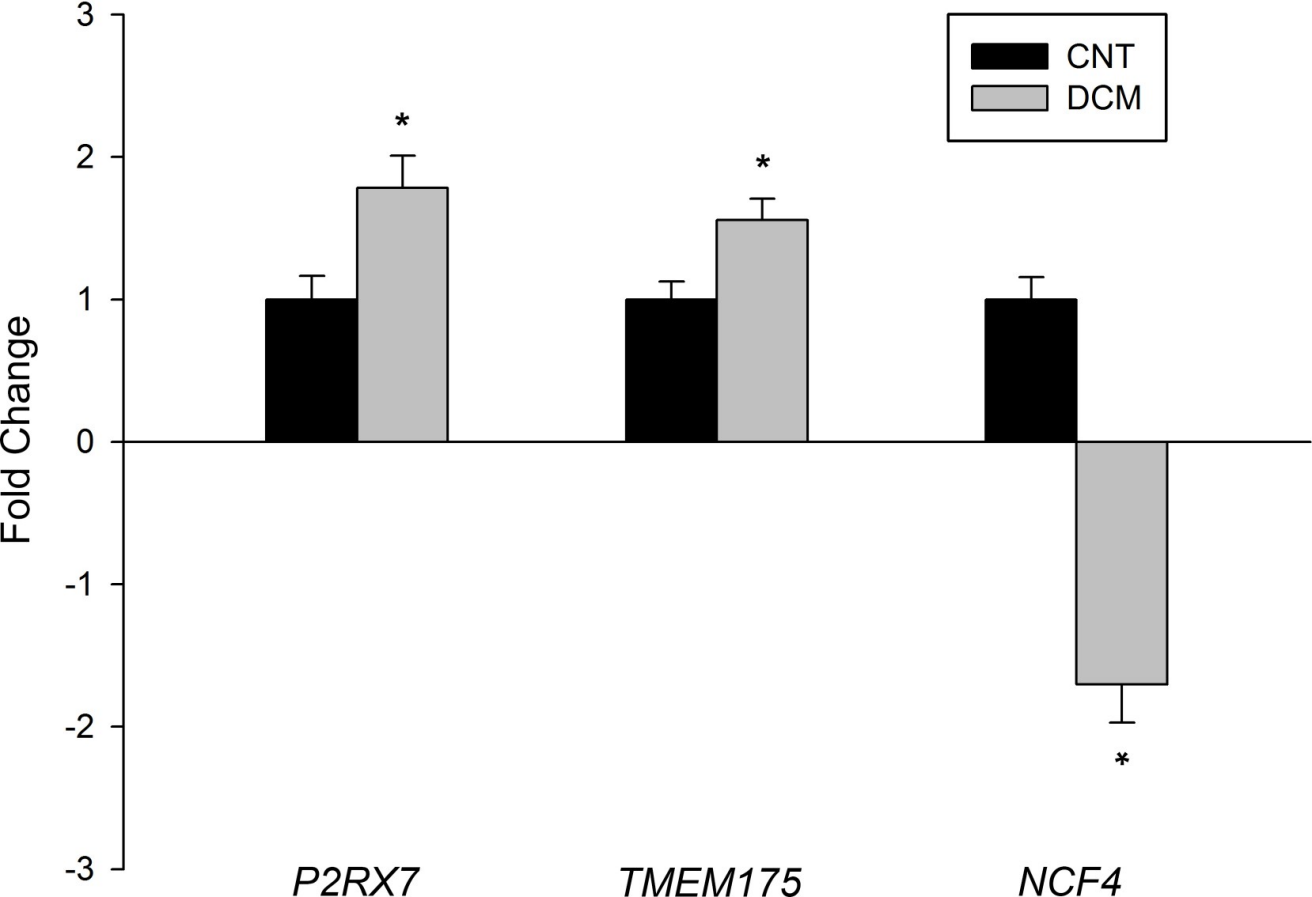
Altered expression of the phagocytosis machinery in dilated cardiomyopathy. Changes in the mRNA expression of phagocytosis-related genes in dilated hearts (gray bars) *vs*. control (CNT) hearts (black bars). The values from the CNTs were set to 1. Bars display the fold change ± standard error of the mean. *P < 0.05 *vs*. CNTs.

With regard to phagocytosis, we identified 44 genes related to this degradation process (S2 Table). Among this gene cluster, the mRNA expression levels of the P2X purinoceptor 7 (*P2RX7*) and transmembrane protein 175 (*TMEM175*) genes were significantly upregulated (≥1.3-fold; *P* < 0.05), whereas those of neutrophil cytosolic factor 4 (*NCF4*) gene were significantly downregulated (≥1.3-fold decrease; *P* < 0.05) in the patients with DCM (Fig 2).

### Relationships between gene expression and function and remodeling parameters

Next, we investigated the potential relationships between the altered mRNA expression levels of autophagy- and phagocytosis-related genes and the clinical parameters of the DCM group. Interestingly, we observed significant correlations between *NRBP2* and the cardiac function and remodeling parameters (*NRBP2 vs*. EF, r = –0.618, *P* < 0.05; *NRBP2 vs*. LVESD, r = 0.638, *P* < 0.05; *NRBP2 vs*. LVEDD, r = 0.645, *P* < 0.05). Furthermore, *CALCOCO2* showed significant negative correlations with both LV end-systolic and end-diastolic diameters (*CALCOCO2 vs*. LVESD, r = –0.588, *P* < 0.05; *CALCOCO2 vs*. LVEDD, r = –0.621, *P* < 0.05) as well as a near-significant correlation with EF (*CALCOCO2 vs*. EF, r = 0.550, *P* = 0.05). The expression levels of *NRBP2* and *CALCOCO2* were in turn inversely correlated (*NRBP2 vs*. *CALCOCO2*, r = –0.787, *P* < 0.001).

### Western blot analyses

**Fig 3:**
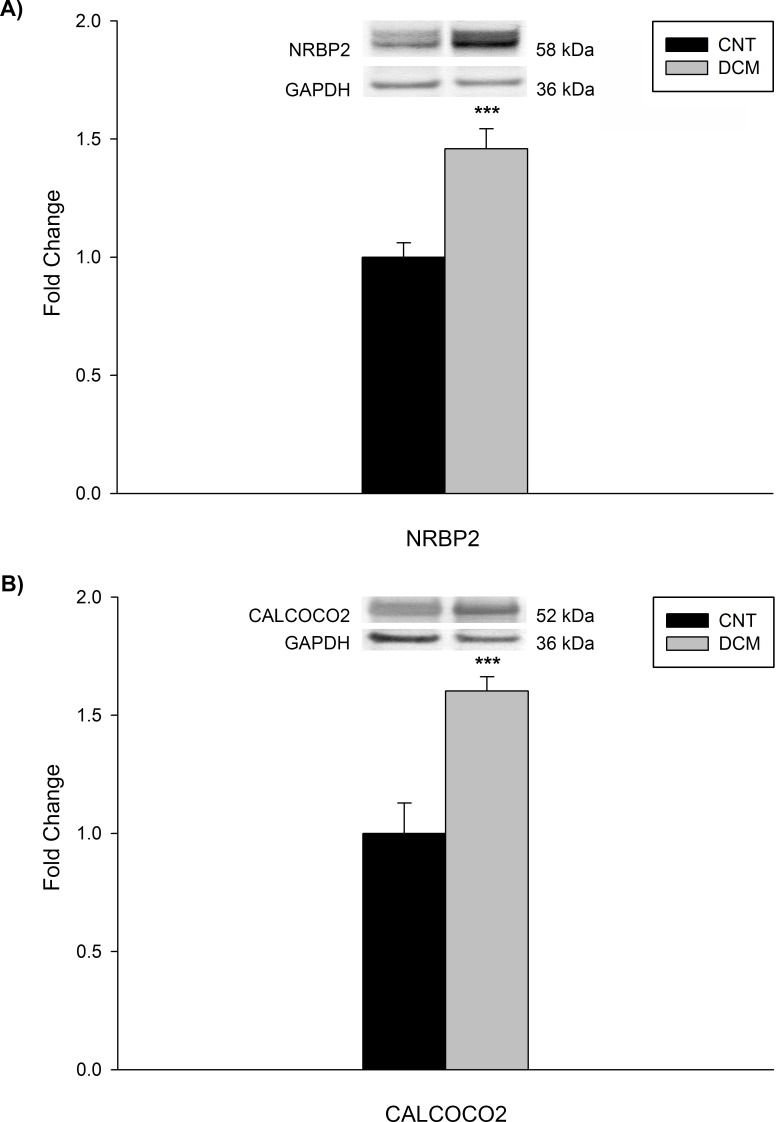
Protein expression levels of NRBP2 and CALCOCO2 in dilated cardiomyopathy. Bar graphs comparing the NRBP2 (A) and CALCOCO2 (B) protein levels in dilated hearts (gray bars) *vs*. control (CNT) hearts (black bars). The values from the CNTs were set to 1. Bars display the fold change ± standard error of the mean. *P < 0.001 *vs*. CNTs.

To determine whether the altered gene expression triggers changes in the protein levels, we performed western blot analysis of NRBP2 and CALCOCO2. We focused on these genes because of its significant association to ventricular dysfunction parameters in patients with DCM. We found that the protein levels of NRBP2 and CALCOCO2 (Fig 3) were indeed significantly increased in these patients relative to the CNT levels [145.88 ± 36.68 *vs*. 100 ± 15.97 arbitrary units (au), *P* < 0.001; 160.22 ± 27.29 *vs*. 100 ± 33.94 au, *P* < 0.001, respectively]. We also observed a significant correlation between both proteins (NRBP2 vs. CALCOCO2, r = 0.583, P < 0.05).

### Ultrastructural analysis of myocardial tissue in DCM

**Fig 4:**
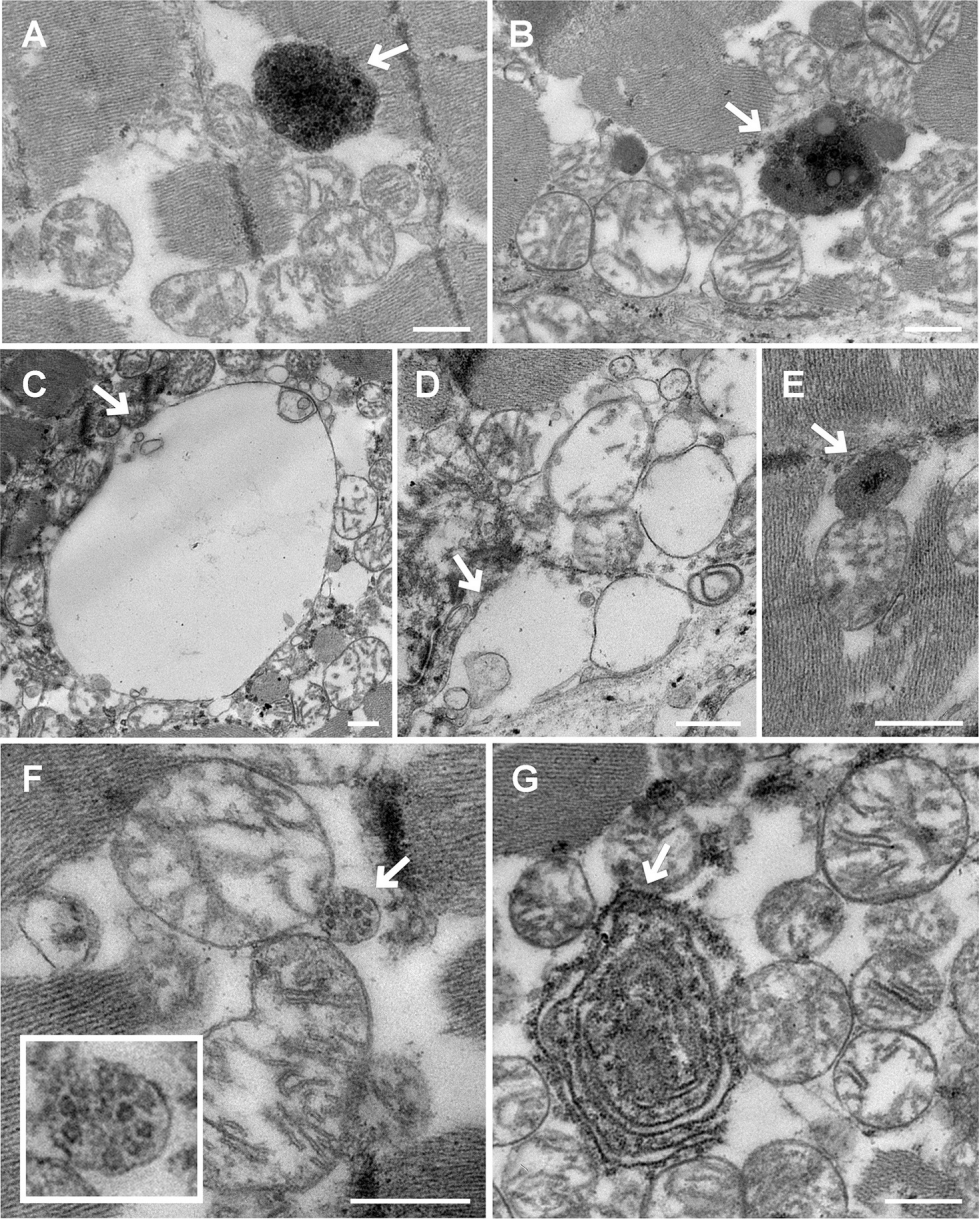
Micrographs of autophagic structures in sections of left ventricular tissue. **A** and **B)** Dense bodies in the control (CNT) tissue and dilated cardiomyopathy (DCM) tissue, respectively. **C)** Vacuolar autophagic structure. **D)** Endosomal structure. **E)** Autophagic structure in the advanced stage (late endosomal + lysosome). **F)** Multivesicular body. **G)** Lamellar or myelinated body. Except for image **A,** the rest of the images are from DCM samples. Arrows indicate the structures of interest. The white bars represent 500 nm.

Multiple structures are related to the cell recycling and degradation processes; namely, dense bodies, autophagic structures, multivesicular bodies, and lamellar structures. Using TEM technology, we observed the presence of dense bodies in the CNT tissue (Fig 4A). In comparison, such dense bodies and autophagic structures (Fig 4B, 4C, 4D and 4E) were increased by 3.19 times in the samples from the patients with DCM (1.66/10 μm^2^ *vs*. 0.52/10 μm^2^, *P* < 0.001). Furthermore, multivesicular bodies (Fig 4F) and lamellar structures (Fig 4G) were present only in the DCM samples.

## Discussion

Degradation pathways are induced in response to external stress, damage, and cell development and remodeling. They are essential for maintaining cellular homeostasis and are required for intracellular self-renewal, energy replenishment, and substrate recycling. Deregulation of the degradation pathways has been linked to various diseases, such as cancer, neurodegenerative disorders and heart failure [16]. However, the functional role of autophagy in heart diseases remains unclear. Regulation of autophagy has been related to both protective and detrimental roles in cardiac cells [17]. Increased autophagy has been found in hearts after ischemia and reperfusion. It has been suggested that upregulation of autophagy provides protection against cell death and improves cardiac function [18–20]; the degradation of proteins and organelles may maintain ATP production and energy homeostasis during ischemia, thus promoting survival of cardiac cells [21]. Nevertheless, other studies have shown that increased autophagy in hypertrophied hearts might mediate the transition from stable cardiac hypertrophy to decompensated heart failure [22]. In support of this, Matsiu et al. reported that the induction of decreased levels of autophagy in a mice model resulted in reduced apoptosis and infarct size compared to the wild type [21]. In this regard, our study showed a new link between deregulation of the degradation pathways and cardiac dysfunction in patients with dilated cardiomyopathy.

We found altered expression of 13 genes involved in the regulation of autophagy including both *CALCOCO2* and Smith-Magenis syndrome chromosome region, candidate 8 (*SMCR8*), which are both genes that participate in most of the autophagy stages. *CALCOCO2* encodes a protein that targets bacteria, cell structures, and particles for their engulfment by autophagosomes [23] for cell recycling and pathogen degradation, thus regulating the process of autophagosome maturation [24]. *SMCR8*, was reported in a recent study to be part of a negative regulatory complex of the initiation step of autophagy [25,26]. In addition to their role as regulators, these genes are directly involved in the process of autophagolysosome development [25].

Furthermore, the mRNA expression levels of the lysosomal protein transmembrane 5 (*LAPTM5*), ubiquitin C-terminal hydrolase L1 (*UCHL1*), and receptor-interacting serine/threonine kinase 2 (*RIPK2*) genes were markedly upregulated (by ≥1.8 fold) in the DCM tissue compared with the CNT tissue levels. Although *LAPTM5* is known to have functional roles in both embryogenesis and hematopoietic cells maturation [27], its specific role in cardiac autophagy is still unknown. *UCHL* is postulated to interact with the lysosomal-associated membrane protein 2 (*LAMP2*) gene as a component of the chaperone-mediated autophagy process [28]. *RIPK2* is an essential regulator of the autophagy mediated via extracellular signal-regulated kinase (ERK) activation, where it was shown by Wex et al. [29] that inhibition of *RIPK2* and ERK altered the process of autophagosome development.

We investigated the potential relationships between the altered mRNA expression levels of autophagy- and phagocytosis-related genes and the clinical parameters of DCM, whereupon we observed significant correlations between *CALCOCO2* and *NRBP2* expression and function and remodeling parameters. As mentioned before, *CALCOCO2* participate in most of the autophagy stages, thus regulating the process of autophagosome maturation. In this study, we found the altered *CALCOCO2* expression at the gene and protein level. Regarding *NRBP2*, its overexpression has been shown to be related to the inhibition of self-renewal processes in carcinoma cells [30]. *NRBP2* also has sequence similarities to *NRBP1*, which plays an important role in intestinal cell homeostasis and interacts with key components of the ubiquitination machinery [31]. Ubiquitination and autophagy have recently been highlighted as having collaborative roles [32,33]. Although less is known about the functions of *NRBP2*, we found that its overexpression was related to diminished ventricular function in patients with DCM. The altered expression of *NRBP2* was translated into changes at the protein level. The increased levels of *NRBP2* may alter intracellular self-renewal and the recycling of components, resulting in damage of the autophagy process and the disruption of cellular homeostasis. *NRBP2* expression was related to *CALCOCO2*, which is involved in different stages of the autophagy process, and was in turn associated with altered remodeling parameters. The correlations found between both *NRBP2* and *CALCOCO2* at the gene and protein level may reflect some type of regulation between both molecules. Therefore, these changes in expression of autophagy-related genes may considerably alter the autophagy machinery, preventing the proper maturation and function of autophagosomes and autophagolysosomes and producing serious cellular damage.

With regard to phagocytosis (the other main degradation process), we also observed altered mRNA expression levels of some related genes in patients with DCM compared with the CNT levels. Both *P2RX7* and *TMEM175* were significantly upregulated whereas *NCF4* was downregulated in the patients with DCM. *P2RX7* is essential for the maturation of phagosomes and phagolysosome assembly [34–36]. *TMEM175* was reported by Cang et al. [37] to be involved in the phagosome-lysosome fusion process of phagolysosome formation. *NCF4* has an important role in the defense against pathogens. The protein encoded by this gene is part of the NADPH oxidase complex; *NCF4* participates in reactive oxygen species-induced phagocytosis in neutrophils, and mutations in this gene have been described as a cause of chronic granulomatous disease due to defects in NADPH oxidase [38,39]. Thus, the differential expression of these genes may affect phagocytosis (mainly the phagolysosome assembly), leading to alteration of the process function and the decreased viability of cardiomyocytes, thereby worsening the status of patients with DCM.

### Ultrastructural analysis of myocardial tissue in DCM by transmission electron microscopy

In previous studies, we reported structural alterations in the Golgi apparatus of patients with DCM and changes in the expression of genes related to Golgi function. We found that the Golgi vesicles were smaller, more ellipsoidal, and higher in number in DCM, corresponding to a high vesicular density and secretion rate [6]. This organelle has been described as a potential source for autophagosome development [9], which is an essential stage of the autophagy process. Taking these results into account, we wanted to analyze potential alterations in the autophagic structures.

Autophagic vacuoles (a morphological term that describes autophagosomes or autophagolysosomes), have been observed in cardiomyocytes by electron microscopy [40,41]. Saito et al. [40] defined autophagic vacuoles as being double-membrane bodies of variable size (1–10 μm), containing glycogen granules, ribosomes, damaged mitochondria, and other organelles.

In this study, compared with the CNT tissue, the DCM tissue samples showed a greater number of autophagic structures that provide evidence of increased tissue repair and cellular recycling mechanisms. A high density of Golgi vesicles may better supply the cellular material required for autophagosome and phagosome development in the respective degradation processes. Furthermore, multivesicular bodies and lamellar structures were not observed in any of the CNT samples, being specific to DCM. Damage to both autophagy and phagocytosis results in the alteration of intracellular self-renewal and component recycling, and the disruption of cell homeostasis. Hence, our findings suggest new targets of the cellular degradation machinery that should be further investigated in order to elucidate the roles of autophagy- and phagocytosis-related genes in modulating cardiac remodeling and LV dysfunction. A clearer understanding of these molecular mechanisms may lead to new therapeutic options for patients with DCM.

### Strengths, limitations and implications of the study

To ensure that our study population was etiologically homogeneous, we chose patients with DCM who did not report a family history of the disease. In this study, a significant number of human pathological and CNT samples were used, allowing our results to be extrapolated to other populations with DCM. In addition, our presence in the operating room at the time of transplantation allowed us to obtain specific regions of cardiac tissue for the analyses performed, which would not have been possible had we used endomyocardial biopsies as samples. This procedure also ensured the optimum preservation of the samples. One limitation of this study was the inherent variability of human cardiac samples. Although all patients were receiving conventional treatment, some therapies might have influenced our results.

## Conclusions

We have found the altered expression of a total of 16 genes involved in both the autophagy (13 genes) and phagocytosis (3 genes) degradation processes. The changes in *NRBP2* and *CALCOCO2* expression were associated with cardiac dysfunction and remodeling. Through TEM, we observed increased tissue repair and cellular recycling mechanisms in DCM, as evidenced by the greater number of autophagic structures in the diseased tissue relative to the CNTs. Furthermore, the multivesicular bodies and lamellar structures observed were specific to DCM. Our findings suggest new targets of the cellular degradation machinery that could be used for the development of therapies to improve LV performance.

## Supporting information

S1 Table Additional data on autophagy-related genes expressed in patients with dilated cardiomyopathy. (XLSX)

S2 Table Additional data on phagocytosis-related genes expressed in patients with dilated cardiomyopathy. (XLSX)
